# Case Report: Biweekly TAS-102 and bevacizumab as maintenance therapy for recurrent and metastatic pseudomyxoma peritonei following palliative cytoreduction

**DOI:** 10.3389/fonc.2025.1631286

**Published:** 2025-12-15

**Authors:** Baoxuan Wang, Yan Wang, Dong Zhao, Houjun Zhang, Qi Liu, Guanying Yu, Jie Jiao

**Affiliations:** 1 Department of Gastrointestinal Surgery, Central Hospital Affiliated to Shandong First Medical University, Jinan, China; 2 Department of Endocrinology, Shandong Provincial Hospital, Affiliated to Shandong First Medical University, Jinan, China

**Keywords:** pseudomyxoma peritonei (PMP), preoperative preparation, pulmonary metastasis, lymphatic metastasis, chemotherapy regimen

## Abstract

**Introduction:**

Recurrence of pseudomyxoma peritonei (PMP) with pulmonary and lymphatic metastases occurs only rarely in clinical practice, making the selection of an appropriate postoperative chemotherapy regimen essential for managing these complex cases.

**Case presentation:**

We describe an 83-year-old woman with an appendiceal pseudomucinous tumor who developed a recurrence 2 years after initial surgery, presenting with bilateral lung metastases and multiple lymph node metastases. Palliative cytoreductive surgery (CRS) combined with hyperthermic intraperitoneal chemotherapy (HIPEC) was performed to relieve intestinal obstruction. Given the patient’s advanced age and the complexity of the procedure, the completeness of the cytoreduction score was 2, and the peritoneal cancer index (PCI) was 10. Postoperative pathology confirmed a high-grade mucinous adenocarcinoma of appendiceal origin. After initiating a biweekly regimen of trifluridine/tipiracil (TAS-102) combined with bevacizumab, the patient achieved disease stabilization for approximately 5 months, with stable tumor marker levels and no evidence of radiological progression.

**Conclusion:**

This case underscores the therapeutic challenges of recurrent PMP with bilateral pulmonary and multiple lymph node metastases. A biweekly regimen of TAS-102 plus bevacizumab may represent a viable maintenance option when surgery and conventional chemotherapy regimens prove unsatisfactory.

## Introduction

Pseudomyxoma peritonei (PMP) is a rare disease with a reported annual incidence of approximately 1–3 cases per million (1). Cytoreductive surgery (CRS) combined with peritoneal hyperthermic intraperitoneal chemotherapy (HIPEC) is considered to be an effective treatment to improve disease control and enhance long-term survival (2). However, patients who fail to achieve complete tumor cytoreduction have a significantly poorer prognosis compared to those with complete or optimal tumor cytoreduction (3). Pulmonary and lymphatic metastases are relatively rare occurrences in PMP, and its primary metastatic pathway is intra-abdominal implantation.

We report a case of recurrent PMP with lung and lymph node metastases, palliative surgery, and a limited response to multiple chemotherapy regimens, which collectively presented substantial therapeutic challenges. This case illustrates the recurrence patterns and metastatic pathways of PMP and describes an effective chemotherapy regimen used at our center, offering a useful reference for the future management of similarly complex presentations.

## Case presentation

**Figure 1:**
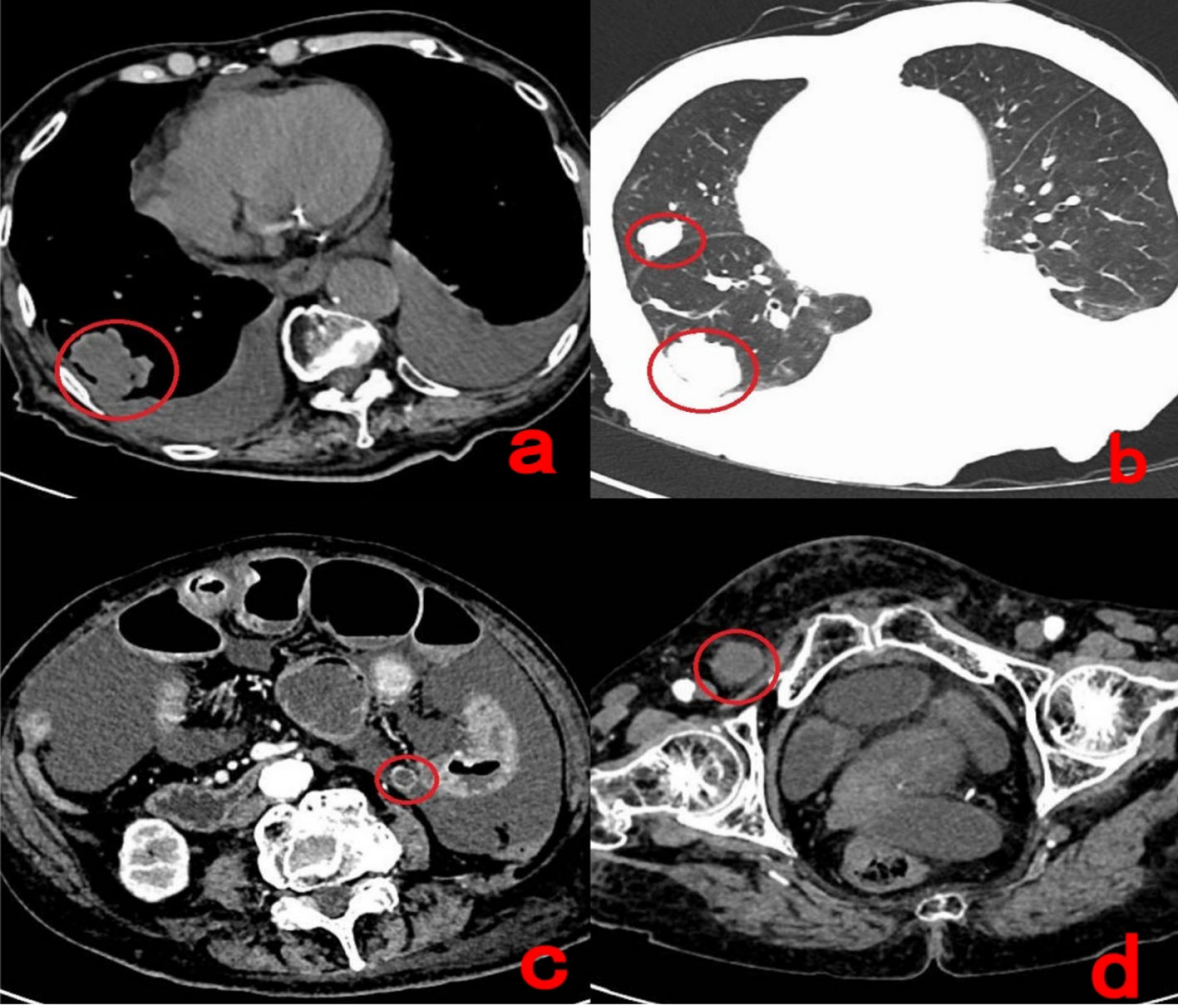
**(a, b)** Multiple soft tissue density nodules and masses **(b)** were scattered throughout both lungs; the largest of these, located in the right lower lobe's outer basal segment **(a)**, exhibited lobulated margins and demonstrated mild enhancement on post-contrast imaging. **(c)** Image c reveals retroperitoneal lymphadenopathy with heterogeneous enhancement following contrast administration. **(d)** Multiple enlarged lymph nodes were also present in the bilateral inguinal region, showing mild, heterogeneous enhancement on the contrast scan, as indicated by the red circles.

An 83-year-old woman presented with worsening ascites and abdominal distension more than 2 years after surgery for pseudomyxoma peritonei. Her initial right hemicolectomy had revealed a high-grade mucinous adenocarcinoma with a pathological stage of pT4aN1M0. She was originally diagnosed with appendiceal pseudomyxoma peritonei at another institution, where she underwent a right hemicolectomy followed by 13 cycles of bevacizumab plus capecitabine. After a marked elevation in tumor markers, her treatment was changed to TAS-102 combined with bevacizumab (TAS-102–35 mg/m^2^ orally twice daily on days 1–5 and 15–19, every 4 weeks; bevacizumab 400 mg every 4 weeks). This regimen induced severe myelosuppression, leading to a subsequent switch to a single cycle of bevacizumab with irinotecan. Severe adverse effects, including vomiting and diarrhea, necessitated a return to maintenance therapy with bevacizumab and capecitabine. Although chemotherapy was tolerated, her abdominal distension continued to worsen, accompanied by lower abdominal pain and vomiting. On admission, physical examination revealed abdominal distension, a roughly 20-cm longitudinal surgical scar in the right lower quadrant, and signs of ascites with positive shifting dullness. Laboratory tests showed elevated tumor markers: carcinoembryonic antigen (CEA) at 235.8 ng/mL (normal < 5 ng/mL), CA125 at 88.53 U/mL (normal < 35 U/mL), and CA199 at 5,645 U/mL (normal <37 U/mL). CT imaging identified multiple metastatic nodules in both lungs, the largest measuring 39.7 × 28.5mm in the right lower lobe, which exhibited mild enhancement and lobulated margins. A biopsy of the lung lesions was performed to confirm their metastatic origin. Multiple enlarged lymph nodes were observed in the abdominal cavity, retroperitoneum, and bilateral inguinal regions, with the largest in the right inguinal area measuring 20mm in short-axis diameter and demonstrating mild heterogeneous enhancement. Additionally, marked dilation of small bowel loops was evident, particularly in the left abdomen, with multiple air-fluid levels and enhancing wall thickening suggestive of intestinal obstruction (**Figure 1**). Given her advanced age and prior surgical history, abdominal CT demonstrated marked small-bowel dilatation consistent with intestinal obstruction. The patient was kept nil per os (NPO) and underwent nasogastric tube decompression. Supportive therapy included intravenous proton pump inhibitor, lipid emulsion injection (C6–24), and human serum albumin infusion. After 3 days of conservative management, her abdominal distension showed no significant improvement.

**Figure 2:**
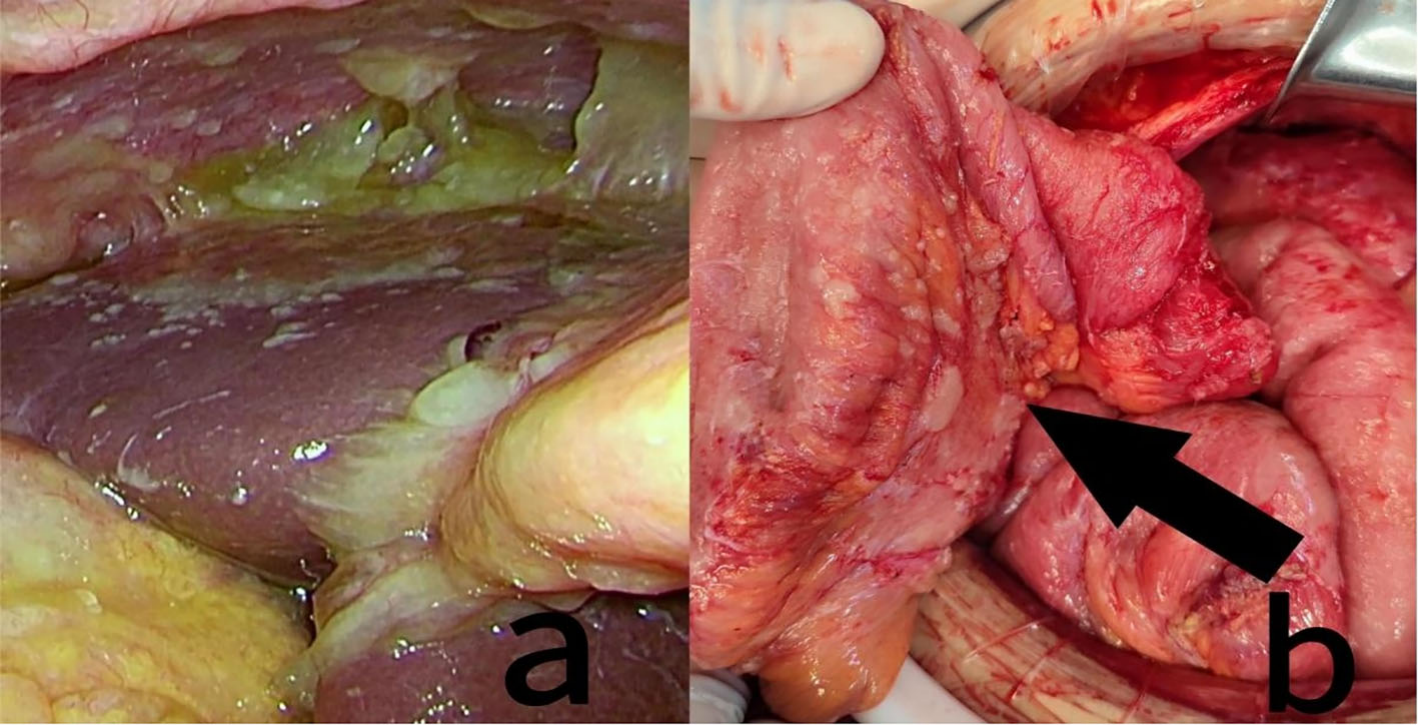
**(a)** Intra-abdominal mucinoid tumors on laparoscopic exploration. **(b)** Intraoperative myxoid tumors were seen to invade small bowel segments (indicated by the black arrow).

**Figure 3:**
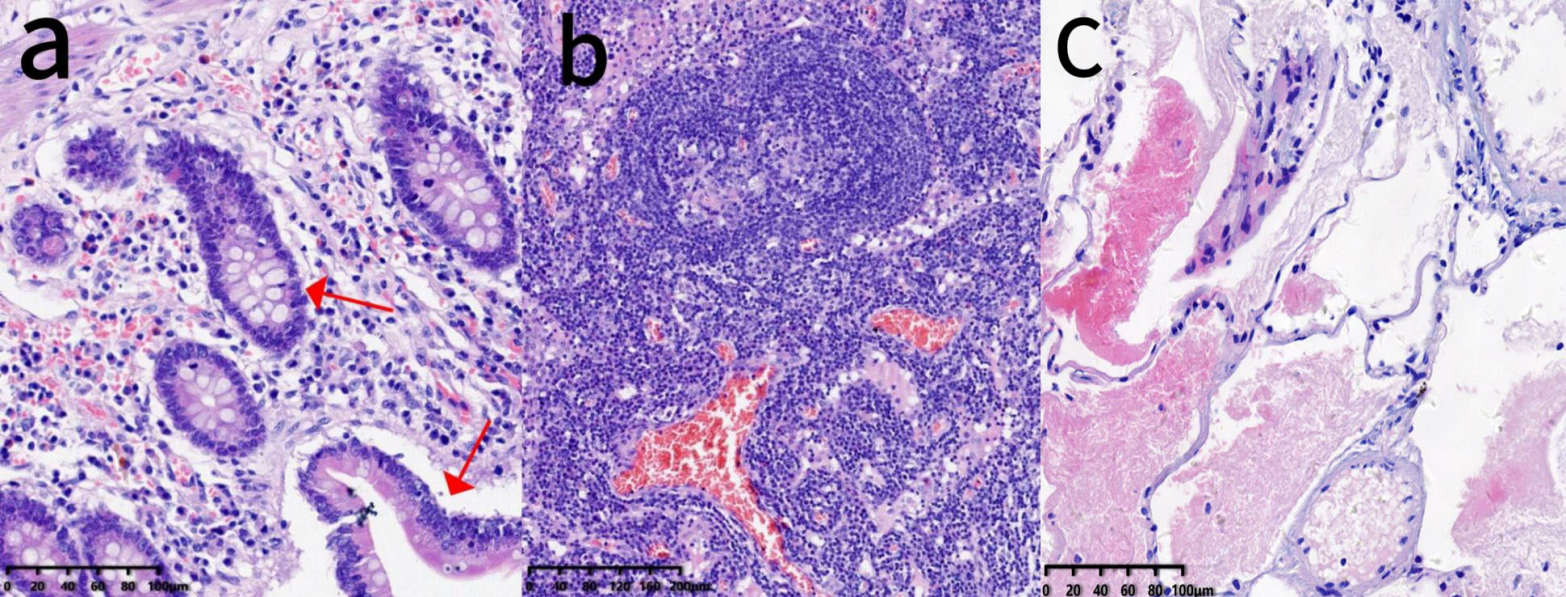
Histopathological and immunohistochemical analysis. **(a)** Metastatic appendiceal mucinous adenocarcinoma cells were identified on the serosal surface of the resected small intestine and mesentery (H&E, ×100) (red arrows). This low-power magnification reveals the architectural pattern of tumor infiltration into the serosal and mesenteric tissues. **(b)** Metastatic carcinoma was present within the peri-intestinal lymph nodes (H&E, ×200) (red arrows). The higher magnification enables detailed observation of the cytological features of the metastatic mucinous adenocarcinoma cells in the lymph node parenchyma. **(c)** Histopathological examination of the preoperative needle biopsy from the pulmonary metastasis confirmed metastatic mucinous adenocarcinoma (H&E, ×100). The ×100 magnification demonstrates the histological structure of this metastatic focus in the lung parenchyma, which is consistent with the primary appendiceal tumor.

**Figure 4:**
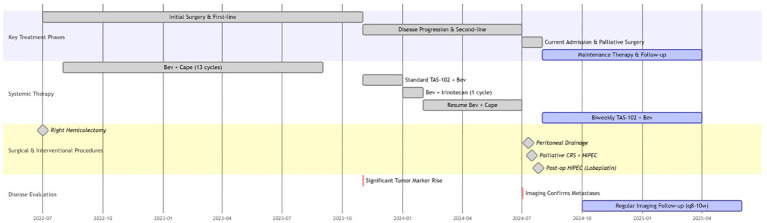
Treatment Timeline. This schematic illustrates the chronological sequence of therapeutic interventions from July 2022 to April 2025. Key phases include initial surgery with first-line therapy, disease progression with multiple salvage regimens, palliative cytoreductive surgery (CRS) plus hyperthermic intraperitoneal chemotherapy (HIPEC), and maintenance therapy. Specific systemic therapies shown are bevacizumab plus capecitabine (Bev+Cape), TAS-102 plus bevacizumab, and surgical procedures including right hemicolectomy. Critical disease evaluation timepoints - significant tumor marker elevation and imaging-confirmed metastases - are indicated, with ongoing imaging follow-ups every 8-10 weeks. The biweekly TAS-102 plus bevacizumab maintenance regimen resulted in 5 months of disease stabilization.

A consultation with the Interventional Oncology Department was obtained, and under ultrasound guidance, peritoneal drainage catheters were placed in both the left and right abdominal cavities. On the day of catheter insertion, 1,000 mL of yellow, viscous ascites was drained from the left side and 75 mL from the right. On postoperative day 2, the drainage volumes were 200 mL (left) and 10 mL (right); on day 3, the volumes were 730 mL (left) and 20 mL (right). By day 4, no further ascites could be drained, most likely due to catheter occlusion caused by disseminated tumor deposits and gelatinous mucinous ascites. Following a multidisciplinary discussion, the patient underwent palliative cytoreductive surgery (palliative CRS) combined with HIPEC. The patient initially underwent laparoscopic exploration, during which approximately 1,000 mL of turbid, mucinous ascites and diffuse peritoneal tumor deposits were observed (**Figure 2a**). The small bowel was markedly dilated (*d* ~ 6cm), edematous, and densely adherent, with multiple mesenteric mucinous nodules; due to technical difficulty, the procedure was converted to open laparotomy. Exploration revealed a stenotic terminal ileal segment adherent to the retroperitoneum, a plaque-like omental mass (15 × 10cm), and dense adhesions around the previous ileocolic anastomosis. Because the duodenal bulb could not be separated, a segment of ileum with severe tumor invasion was resected, and the specimen was removed for pathological examination (**Figure 2b**). The abdominal cavity was thoroughly irrigated with warm distilled water, and perfusion catheters were placed for HIPEC. The intraoperative perfusion regimen consisted of 4,000 mL of normal saline with raltitrexed 4 mg, administered at a temperature of 43°C, with a circulation pump flow rate of 400 mL/min and a total intraperitoneal perfusion volume of 3,000 mL for 1 h. Upon completion of HIPEC, a loop ileostomy was constructed using a circular anastomosis technique. The postoperative completeness of cytoreduction (CC) score was 2, and the peritoneal cancer index (PCI) score was 10. Although preoperative CT indicated small bowel dilation and significant ascites—features that can sometimes lead to overestimation of disease burden—the PCI score of 10 was determined during surgical exploration and reflects direct visualization and palpation of peritoneal surfaces. There was no significant discrepancy between radiological suspicion and intraoperative findings regarding the extent of peritoneal involvement. During the first five postoperative days, the patient received antibiotics for infection control, proton pump inhibitors, and intravenous fluids for nutritional support. As she remained clinically stable without discomfort, one session of HIPEC was subsequently administered. Lobaplatin is a third-generation platinum-based alkylating antineoplastic agent. Its pharmacological characteristics include high water solubility, good stability, a broad anticancer spectrum, and significant antitumor efficacy. Compared to traditional platinum drugs (e.g., cisplatin, oxaliplatin), it demonstrates comparable inhibitory effects on colorectal tumor cells, with relatively milder gastrointestinal reactions and neurotoxicity. The selection of lobaplatin for HIPEC was based on these properties and its demonstrated acceptable safety profile and encouraging efficacy potential in HIPEC regimens for peritoneal metastases, notwithstanding its noted potential effects on platelets and liver function (4). The perfusion regimen consisted of 4,000 mL of normal saline with lobaplatin 50 mg, administered at a temperature of 43°C, with a circulation pump flow rate of 400 mL/min and a total intraperitoneal perfusion volume of 3,000 mL for 1 h. After perfusion therapy, the abdominal drainage tube was removed successively until discharge. The patient did not develop postoperative or post-perfusion complications. The patient was hospitalized for 25 days after surgery. Pathology confirmed high-grade mucinous adenocarcinoma of appendiceal origin with metastasis, as indicated by immunohistochemistry (CK7 [+], CK20 [+], villin [+], Ki-67 [+] with 80% in the hot spot area) and metastatic carcinoma detected in peri-intestinal lymph nodes (**Figures 3a, b**). In addition, a pathological specimen of the pulmonary lesion was obtained by preoperative needle biopsy. Histopathology confirmed the presence of atypical cells in the pulmonary nodules with histological features similar to those of mucinous tumors invading the lung parenchyma (**Figure 3c**). One week after discharge, the patient returned to our hospital for a follow-up visit. Due to failure to achieve complete cytoreduction and adverse reactions associated with the previous chemotherapy regimen, the patient was recommended after a multidisciplinary team (MDT) discussion to initiate maintenance therapy with TAS-102 in combination with bevacizumab. The treatment regimen consists of TAS-102–35 mg/m² orally administered daily on days 1–5 and bevacizumab 200 mg administered every 2 weeks (**Figure 4**). Toxicities were monitored and graded according to CTCAE v5.0. No dose reductions were necessary during the biweekly TAS-102 therapy. At the most recent follow-up, the patient has survived for 5 months since starting the current chemotherapy regimen, with sustained disease control. Serial evaluations conducted during each hospitalization showed stable tumor marker levels (**Table 1**). Disease monitoring included chest and abdominal CT scans performed every 8–10 weeks and assessed using RECIST 1.1 criteria. The patient reported improved abdominal distension and appetite after palliative surgery and the initiation of the new regimen, although no formal quality-of-life instrument was applied. No major treatment-related adverse events were observed. Our medical team will continue to monitor the patient closely.

**Table 1:** The levels of various tumor markers (CEA, CA125, and CA199) remained generally stable during chemotherapy and within normal ranges.

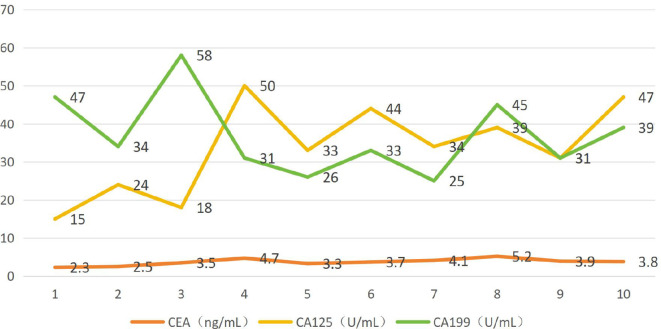

## Discussion

PMP is a rare clinical syndrome with over 95% of cases originating from the appendix and gastrointestinal tract. In the 1990s, Professor Sugarbaker (5) proposed a model for the treatment of PMP by CRS combined with HIPEC, which involves the elimination of residual microscopic lesions and scattered implanted tumor cells in the abdominal cavity by intraperitoneal hyperthermic perfusion chemotherapy after surgical resection of the tumor, thereby reducing tumor recurrence and prolonging the interval between recurrences.

Even though patients with PMP were treated with CRS in combination with HIPEC, the recurrence rate was still high, and in patients with recurrence, reoperation was required to prolong survival (6). Depending on the degree of dissemination and distribution of tumor cells, the appropriate lesions are removed, and finally, based on the degree of completion of CRS, a cytopenic (CC) score is determined postoperatively. Surgery that achieves CC0 or CC1 is termed complete cytoreduction surgery (CCRS). The CC score is closely related to the prognosis of patients with PMP, and even in patients with high-grade PMP, better OS and DFS can be achieved following CCRS (7). Therefore, for most patients with PMP, CCRS should be achieved whenever feasible during surgery. Recurrent PMP poses challenges in removing lesions in some areas due to the disruption of the surgical plane by previous surgery and the formation of adhesions, which may result in tumor cells being retained in the scar tissue. These patients are not suitable for CCRS and may be considered for maximum tumor decrement (MTD). The concept of MTD has been proposed as an alternative to CRS and HIPEC for patients who cannot have their tumor completely removed or are not suitable for prolonged surgery (2). This is also the main reason why the patient underwent palliative CRS + HIPEC.

While pulmonary and lymphatic metastases in PMP are exceedingly rare, our case illustrates an aggressive phenotype with multisystem involvement. The use of biweekly TAS-102 plus bevacizumab represents a tailored approach in a heavily pretreated, elderly patient, differing from conventional regimens and highlighting the need for personalized therapy in such complex scenarios. For PMP, diffuse abdominal implantation dissemination is usually the primary mode of dissemination, while lymphatic and hematogenous metastases are rare. Currently, few cases of appendiceal mucinous adenocarcinoma with pulmonary metastases have been reported. As early as 1964, Berge (8) reported a case of a peritoneal mucinous cyst with pulmonary metastasis. However, to date, the literature in this field is limited to individual case reports, with no large-scale studies further exploring the mechanisms of lung metastasis. Although some reports (9) have suggested that combined HIPEC and HITHOC may be considered in cases of PMP with pleural involvement, in our patient, the thoracic disease was limited to pulmonary nodules without pleural dissemination. Given her advanced age and multiple comorbidities, HITHOC was not selected; instead, palliative CRS + HIPEC and systemic therapy were performed. Regardless of the extent of the tumor, high-grade mucinous peritoneal tumors usually metastasize by direct spread within the peritoneum and rarely through the lymphatic system (10). Compared with previously reported cases of retroperitoneal metastatic tumors, this case demonstrates rare occurrences of pulmonary metastasis and lymph node metastasis. Current research (11, 12) indicates that early detection of metastatic lesions is crucial for disease staging and treatment planning. Imaging-based radiomics technologies and novel biomarkers have been proposed to improve early differentiation between localized lesions and advanced-stage diseases.

In patients with recurrent PMP, especially those with high-grade appendiceal mucinous tumors, the combination of a neoangiogenesis inhibitor (e.g., bevacizumab) with chemotherapy improves the patient’s DFS and OS. This is the reason why bevacizumab has been maintained throughout changes in the patient’s multiline chemotherapy regimen, and in many studies of PMP treatment, 5-fluorouracil-based regimens were used, such as 5-fluorouracil/capecitabine monotherapy, FOLFOX, or FOLFIRI (2). Hirano et al. (13) applied the conventional 4-week TAS-102 combined with bevacizumab regimen to a case of high-grade appendiceal mucinous tumor treated with palliative resection, achieving a high survival rate. TAS-102 is an oral combination agent in which the cytotoxic component trifluridine is incorporated into DNA, inducing DNA strand breaks, while tipiracil inhibits its degradation by thymidine phosphorylase to circumvent key fluoropyrimidine resistance pathways (14). This mechanism differs from the multi-kinase inhibitory activity of regorafenib or rechallenge with FOLFOX, whose efficacy may be constrained by pre-existing drug resistance. Hiroshi Matsuoka (15) and his team concluded in the TAS-CC4 study that a fortnightly dosing regimen reduces hematological adverse effects. The BiTS trial (16) demonstrated that a biweekly TAS-102 regimen combined with bevacizumab reduced hematologic toxicity while maintaining efficacy in advanced colorectal cancer, supporting its use in our elderly patient with prior treatment intolerance. The patient’s systemic therapy was changed multiple times due to progressive disease (rising tumor markers) and treatment-limiting toxicities. Initially, the patient received bevacizumab plus capecitabine (13 cycles); following biochemical progression, she received TAS-102 plus bevacizumab using a conventional 4-week schedule, which produced severe myelosuppression; subsequent single-agent irinotecan with bevacizumab led to intolerable gastrointestinal toxicity. Given limited options and prior hematologic toxicity, we selected a biweekly TAS-102 regimen combined with bevacizumab (reduced dose per-cycle intensity) based on published phase II/III data and case reports indicating comparable disease control with improved tolerability. The BiTS trial supports a fortnightly TAS-102 dosing schedule to reduce hematologic adverse events. Therefore, the choice of this regimen in our patient was driven by 1) prior intolerance to standard schedules, 2) evidence for activity of TAS-102 in refractory gastrointestinal adenocarcinomas, and 3) an individualized risk–benefit assessment in an elderly patient with multiple comorbidities.

## Conclusion

We report a rare case of recurrent appendiceal high-grade PMP with bilateral pulmonary and multiple lymph node metastases, managed with palliative CRS + HIPEC for symptom control. Due to incomplete cytoreduction and prior toxicity from chemotherapy, the patient received a biweekly regimen of TAS-102 plus bevacizumab, which was well tolerated. Although efficacy cannot be determined from a single case, this combination may represent an alternative for selected patients with refractory PMP, pending further prospective data.

## Data Availability

The original contributions presented in the study are included in the article/**Supplementary Material**. Further inquiries can be directed to the corresponding authors.

## Supplementary material

The Supplementary Material for this article can be found on line at: https://www.frontiersin.org/articles/10.3389/fonc.2025.1631286/full#supplementary-material
