# Supplementary material for: Case Report: Biweekly TAS-102 and bevacizumab as maintenance therapy for recurrent and metastatic pseudomyxoma peritonei following palliative cytoreduction
Source: Front Oncol. 2025 Dec 15;15:1631286. doi: 10.3389/fonc.2025.1631286 (PMC12745458; doi:10.3389/fonc.2025.1631286)
Supplement: Supplementary file 1 [file DataSheet1.pdf]

CARE
case report guidelines

CARE Checklist of information to include when writing a case report

(cc) BY-NC-ND

✓

Topic	Item	Checklist item description	Reported on Line
**Title**	1	The diagnosis or intervention of primary focus followed by the words "case report"	√
**Key Words**	2	2 to 5 key words that identify diagnoses or interventions in this case report, including "case report"	√
**Abstract (no references)**	3a	Introduction: What is unique about this case and what does it add to the scientific literature?	√
	3b	Main symptoms and/or important clinical findings	√
	3c	The main diagnoses, therapeutic interventions, and outcomes	√
	3d	Conclusion—What is the main "take-away" lesson(s) from this case?	√
**Introduction**	4	One or two paragraphs summarizing why this case is unique (**may include references**)	√
**Patient Information**	5a	De-identified patient specific information.	√
	5b	Primary concerns and symptoms of the patient.	√
	5c	Medical, family, and psycho-social history including relevant genetic information	√
	5d	Relevant past interventions with outcomes	√
**Clinical Findings**	6	Describe significant physical examination (PE) and important clinical findings.	√
**Timeline**	7	Historical and current information from this episode of care organized as a timeline	√
**Diagnostic Assessment**	8a	Diagnostic testing (such as PE, laboratory testing, imaging, surveys).	√
	8b	Diagnostic challenges (such as access to testing, financial, or cultural)	√
	8c	Diagnosis (including other diagnoses considered)	√
	8d	Prognosis (such as staging in oncology) where applicable	√
**Therapeutic Intervention**	9a	Types of therapeutic intervention (such as pharmacologic, surgical, preventive, self-care)	√
	9b	Administration of therapeutic intervention (such as dosage, strength, duration)	√
	9c	Changes in therapeutic intervention (with rationale)	√
**Follow-up and Outcomes**	10a	Clinician and patient-assessed outcomes (if available)	√
	10b	Important follow-up diagnostic and other test results	√
	10c	Intervention adherence and tolerability (How was this assessed?)	√
	10d	Adverse and unanticipated events	√
**Discussion**	11a	A scientific discussion of the strengths AND limitations associated with this case report	√
	11b	Discussion of the relevant medical literature **with references**.	√
**Patient Perspective**	11c	The scientific rationale for any conclusions (including assessment of possible causes)	√
**Informed Consent**	11d	The primary "take-away" lessons of this case report (without references) in a one paragraph conclusion	√

12 The patient should share their perspective in one to two paragraphs on the treatment(s) they received . . . . ___ √_________________

13 Did the patient give informed consent? Please provide if requested . . . . **Yes** ☑ **No** ☐
